# County Nursing Associations and Registration

**Published:** 1920-01-03

**Authors:** 


### THE MATRONS' AND SISTERS' DEPARTMENT.

### COUNTY NURSING ASSOCIATIONS AND REGISTRATION.

In the debate which marked the second reading of the Bill for the State Registration of Nurses, Dr. Addison firmly refused to allow an amendment which would in effect have instituted a second order of nurses, that of the partially trained. General satisfaction was felt at this firm stand on the part of the Health Minister, for it was recognised that the principle of a three years' general training was at stake. Were that bulwark once overthrown, and the public to become aware that an inferior kind of nurse was to be had under the ægis of registration, great harm would be done to the profession. Few lay people would understand how very necessary it is for the patient to be under the care of a skilled woman. Patients' friends are only too prone to regard the nurse as a person whose main duty it is to save them trouble, and a very slight knowledge of the cases which are represented by relatives as "very light" will convince the most sanguine that there would be a general rush for imperfectly trained women at lower rates if only the Registration Board would be obliging enough to give them a *cachet*.

Dr. Addison spoke with no uncertain voice in declining the proposal to introduce pseudo nurses to the register by a back door. But the matter is not so simple as it seems. We must again at the risk of repetition call attention to the wholesale encouragement given to untrained nurses which is going on all over the country at the instance of public bodies, subsidised with State grants. The open door is found in training centres maintained by the County Associations. All over the country in small towns and villages there is an acute demand for midwives, and the County Associations are doing their best to find probationers willing to undergo a short course. They prepare these woman for the C.M.B. examination, despatch them to the various centres which are crying out for them and keep them under inspection. So acute is the demand for this kind of help in some counties that almost any woman who holds the C.M.B. certificate is certain of a good post as "District nurse," and can be at once installed in a position where she will have to undertake general cases often many miles from a doctor. Few committees ask what qualifications the midwife possesses, and if they do she will produce, perhaps, a letter from some chronic patient she once attended on for a few months, or at best a formal letter from a doctor declaring he was satisfied with her on such or such an occasion. Those who can show they have worked for six months at the County Home, entirely dissociated, be it understood, from any kind of hospital, get the prizes in this competition for midwives with "general training." Midwives who thus launch themselves into the nursing world through the County Associations can command salaries amounting, with allowances, to £130 and more a year. How easy it is for them to slide after this into private practice all acquainted with the facts can grasp.

How can the public ever be made to understand that these are not "nurses" in any real sense? Every one addresses them as "nurse." Their dogmatism about every disease and every form of treatment passes as inspired. Country doctors must depend on them for help in operations. For the most part they are hardworking and very worthy women. But we reiterate they are not nurses. Are they to continue, after registration has become a *fait accompli*, to bear the same title as registered nurses, only without the mystic word "registered" which no one will ever dream of using? How are country folk, rich or poor, to discriminate between ignorant "Nurse Smith" and properly trained "Nurse Smith, R.N."?

The Health Minister cannot ignore the process through which these untrained nurses are rapidly increasing in number, for he has to sanction grants made to the county and local nursing associations employing them. Dr. Addison will not have them on the register, and in that we are sure he is right. But is he aware of the hopeless inconsistency of granting money to enable midwives to pose as trained nurses, while so much stress is being laid on the importance of sound training? A liberal system of grants to the Jubilee Institute would enable that body to stimulate the production of twice as many real district nurses as it can now supply. The women who now make haste to be "nurses" and think six months sufficient, are wanted as probationers in the under-staffed hospitals all over the country, and if the short cut were not so easy and lucrative might become real nurses. It is mischievous to encourage them to believe that they can undertake any kind of medical and surgical case after a few weeks or months spent in going round a district with the superintendent. If we must set midwives to do general nursing during a transition time, let us take heed we secure a higher title for the registered than that which has been appropriated by the untrained. Either limit them to the use of the word "midwife" or confer the title of "nursing sister" on registered nurses.

